# Molecular Characterization and Expression of *OfJNK* and *Ofp38* in *Ostrinia furnacalis* (Guenée) Under Different Environmental Stressors

**DOI:** 10.3389/fphys.2020.00125

**Published:** 2020-02-25

**Authors:** Li Su, Jian-Yu Meng, Hong Yang, Chang-Yu Zhang

**Affiliations:** ^1^Provincial Key Laboratory for Agricultural Pest Management of Mountainous Regions, Institute of Entomology, Guizhou University, Guiyang, China; ^2^Guizhou Tobacco Science Research Institute, Guiyang, China

**Keywords:** *Ostrinia furnacalis*, c-Jun N-terminal kinase, p38 mitogen-activated protein kinase, gene cloning, expression analysis, environmental stressors

## Abstract

*Ostrinia furnacalis*, an important pest of corn, has substantial detrimental effects on corn production. The mitogen-activated protein kinase (MAPK) signaling pathway plays a pivotal role in an insect’s resistance to environmental stress. The expression levels of *JNK* and *p38* have been well recorded in several insects under different environmental stressors, at different developmental stages, and in various tissue types; however, there is limited information on *JNK* and *p38* in agricultural insects. To clarify the mechanism whereby *O. furnacalis* responds to environmental stress, we cloned *JNK* and *p38* from *O. furnacalis* and subsequently named them *OfJNK* and *Ofp38*, respectively. Further, we examined the expression levels of *OfJNK* and *Ofp38* under different environmental stressors. In this study, we obtained full-length sequences of *OfJNK* and *Ofp38*, and RT-qPCR results showed that these genes were expressed at all developmental stages, in various tissues (head, chest, abdomen, leg, wing, antennae, compound eye, midgut, and ovary) and under different environmental stressors (4°C and ultraviolet A treatment for 0, 30, 60, 90, and 120 min). The expression levels of *OfJNK* and *Ofp38* were relatively higher in eggs and 3-day-old adult females than in other developmental stages. Moreover, the expression level of *OfJNK* was higher in the wings than in other tissues, whereas that of *Ofp38* was significantly higher in the ovaries than in other tissues. *OfJNK* and *Ofp38* showed high expression 90 min after being subjected to treatment at 4°C and ultraviolet A irradiation; the expression of *Ofp38* peaked at 30 min, whereas that of *OfJNK* peaked at 60 min. These results indicate that *O. furnacalis* differs in terms of its response under different environmental stressors. In summary, our results will provide a foundation for additional research needed to determine the role of the MAPK signaling pathway and the underlying mechanisms by which it shows resistance to environmental stresses in insects.

## Introduction

*Ostrinia furnacalis* (Guenée) (Lepidoptera: Pyralidae) is an important global agricultural pest that has caused substantial economic losses in corn, sorghum, cotton, and millet production (Afidchao et al., 2013). Its first and second instar larvae easily damage the whorl leaves of corn and then attack its stalk, ears, and cobs. In addition, the larvae feed on silk and grains, leading to ear rot, increased mycotoxin contamination, and declined corn quality (He et al., 2003). It is estimated that *O. furnacalis* can cause yield losses of 10–30% and might lead to absence of harvest in an outbreak year (Wang et al., 2000). The global harm to crops by *O. furnacalis* is related to its high underlying capacity to survive under various environmental stresses (Zhang et al., 2013; Liu et al., 2014). Several studies have demonstrated that *O. furnacalis* exhibits physiological adaptations that are induced by low temperature and ultraviolet (UV) stress. For instance, Feng et al. (2007) reported that low temperature treatment could increase the cold tolerance in *O. furnacalis*. Shang et al. (2015) demonstrated that the accumulation of glutathione transferase in larvae is most probably involved in a low temperature stress response. Recently, a comparative proteomic study has demonstrated that the response patterns of *O. furnacalis* to UV-A stress are complex because the differentially expressed proteins were involved in diverse biological processes including signal transduction, transport processing, cellular stress response, metabolism, and cytoskeleton organization (Zhang and Meng, 2018). Moreover, our previous study found that UV-A irradiation can enhance the fecundity of *O. furnacalis* and prolong the development of F_1_ generation (Liu et al., in press). However, the molecular mechanisms via which *O. furnacalis* exhibits the remarkable potential to adapt to these stresses remain unclear.

Insects adapt to fluctuant and unfavorable environments (Grubor-Lajsic et al., 1992; Bale and Hayward, 2010). Low temperature stress is a common environmental stimulus for insects (Fujita, 1999). A previous study has shown that the mitogen-activated protein kinase (MAPK) is cold responsive and that in *Microdera punctipennis* exposed to low temperatures, MAPK may play a role in the signal transduction (Meng et al., 2015). Hu et al. (2017) reported that in *O. furnacalis*, the relative expressions of *Hsc70* and *Hsp90* were induced upon exposure to low temperature. The UV-A have been widely used as light sources in light traps for the management of agriculture and forest insect pests which exhibit phototaxis. However, increasing evidence indicates that UV-A irradiation is an important stress factor that impacts all living creatures including insects (Meyer-Rochow et al., 2002; Meng et al., 2009). Ali et al. (2017) found that UV-A could induce oxidative stress and alter antioxidant enzyme activity, thereby affecting the normal physiological functions of *Mythimna separata* adults. Meng et al. (2010) reported that MAPK was upregulated following UV light irradiation in *Helicoverpa armigera*. Insects can apparently perceive signals using sensors and transmit them to the cellular machinery via signal transduction to regulate gene expression. The MAPK signaling pathway is one of the major signal transduction pathways (Meng et al., 2010).

MAPK is a serine/threonine protein that is implicated in the regulation of multiple cellular activities, such as differentiation, proliferation, death, and adaptive and immune responses (Zhang and Liu, 2002; Huang et al., 2009). Multicellular organisms have two well-characterized subfamilies of MAPKs, c-Jun N-terminal kinase (JNK) and p38 (Johnson and Lapadat, 2002), each of which plays a vital role in the physiological processes of cells, including growth and development, apoptosis, and responses to external environmental stresses (Cowan and Storey, 2003). JNK and p38 are pivotal for the stress-related signaling pathways that transport signals from the cell epidermis into the nucleus to initiate gene expression (Rahaus et al., 2004). JNK is mainly implicated in the transcriptional regulation of cells, and the activation of p38 is generally believed to be a response of cells to inflammatory cytokines and environmental stresses [e.g. UV irradiation, temperature, ischemia, and hypoxia] (Ono and Han, 2000; Hohmann, 2002). Several studies have suggested that JNK and p38 participate in cellular responses to environmental stresses (Xia et al., 1995; Ludwig et al., 2003; Vogelweith et al., 2011).

Several important roles of the MAPK signaling pathway have been reported in insects. For example, Fujiwara and Denlinger (2007) showed that p38 might be part of the signaling pathway that causes rapid frozen stiffening in the flesh fly *Sarcophaga crassipalpis*. Furthermore, their studies on *Bombyx mori* revealed an underlying function of MAPKs of initiating and terminating embryonic diapause (Fujiwara et al., 2006). Moreover, it has recently been demonstrated that the JNK and p38 MAPK signaling pathways play significant roles during the responses of *Bemisia tabaci* to low temperature stresses (Li et al., 2012; Wang et al., 2013). Nevertheless, until now, information about the function of MAPKs in Lepidoptera under various environmental stresses is lacking. The aim of this study was to investigate whether the MAPK signaling pathway is involved in responses of *O. furnacalis* to different environmental stresses. To determine this, we cloned the *JNK* and *p38* of *O. furnacalis* (subsequently named *OfJNK* and *Ofp38*, respectively), examined their expression levels at various developmental stages and in different tissues, and investigated the expression levels of *OfJNK* and *Ofp38* under low temperature and UVA irradiation. Our results could contribute to identifying the molecular mechanism of MAPKs in *O. furnacalis* under various environmental stresses and to designing cultural management strategies that could be used to control this pest.

## Materials and Methods

### Insect Rearing

*O. furnacalis* were reared in climate-controlled cabinets under a 14:10-h light–dark photoperiod at 26 ± 1°C and 70–80% relative humidity. The larvae were fed an artificial diet as previously described (Feng et al., 2011). Three-day-old adult insects were fed a 10% honey–water solution that was kept in a 100-mL transparent plastic container.

### Insect Treatment and Sample Collection

The experimental insect treatments are as follows: (1) low temperature treatment: female adults aged 3 days old were exposed to a temperature of 4°C for 0 (control), 30, 60, 90, and 120 min, and ten female adults were collected per biological replicate. (2) UVA treatment: female adults aged 3 days old were irradiated with 315–400 nm UVA light (NanJing HuaQiang Electronic Engineering Co., Ltd., Nanjing, China) at a frequency of ∼300 μW/cm^1^. After adapting to a 2-h scotophase period at 26 ± 1°C, female adults aged 3 days old were exposed to UVA for 0 (control), 30, 60, 90, and 120 min, and ten female adults were collected per biological replicate. (3) Samples were collected in different developmental stages: eggs, the first to fifth instar larvae, 3-day-old pupae, and 3-day-old adults (females and males). A total of 60 eggs, 40 first instar, 20 second instar, 15 third instar, 10 fourth to fifth instar larvae, 3-day-old pupae, and 3-day-old adults were collected per biological replicate. (4) nine different tissues, including those from the head (without antennae and compound eye), chest, abdomen, leg, wing (from 10 adults), antennae, compound eye (from 30 adults), midgut and ovary (from 20 adults), were collected from *O. furnacalis* female adults aged 3 days old and were individually dissected on ice in phosphate-buffered saline solution with RNase inhibitor (TaKaRa, Dalian, China) using the Olympus binocular microscope (Germany). *O. furnacalis* female adults aged 3 days old (*n* = 30) were maintained in an incubator at 26°C and used as control (0 min). All samples were quickly frozen in liquid nitrogen and stored at −80°C for subsequent RNA extraction. Each treatment group had three biological replicates.

### Total RNA Extraction and cDNA Synthesis

RNA was extracted from the *O. furnacalis* adults using the Trizol reagent (Invitrogen, Carlsbad, CA, United States) following the manufacturer’s protocol. The 260/280 ratio was measured using the NanoDrop 2000 (Thermo Fisher Scientific, Waltham, MA, United States) to evaluate the purity of the total RNA, and the integrity of the isolated RNA was verified using 1% agarose gel electrophoresis. Reverse transcription was conducted using the RevertAid First-Strand cDNA Synthesis Kit (Thermo Fisher) according to the manufacturer’s instructions. The synthesized first-strand cDNAs were stored at −20°C and used as templates.

### Cloning *OfJNK* and *Ofp38* From *O. furnacalis*

Based on conserved *JNK* and *p38* sequences from *Spodoptera litura*, *Dichocrocis punctiferalis*, and *H. armigera*, degenerate primers were designed to amplify *OfJNK* and *Ofp38* fragments (Table 1). Polymerase chain reaction (PCR) was conducted using the following amplification conditions: initial cycle at 94°C for 3 min, 35 amplification cycles (denaturation at 94°C for 30 s, annealing at 53–57°C for 30 s, and elongation at 72°C for 1 min), and a final elongation step at 72°C for 10 min. Electrophoresis of the PCR products was run on 1% agarose gel. The expected fragments were purified using the SanPrep DNA Extraction Kit [Sangon Biotech (Shanghai) Co., Ltd., Shanghai, China] and cloned into the pMD-18T vector (TaKaRa). The ligation products were then transformed into the *Escherichia coli* DH5α cell line (TaKaRa), and the cloned fragments of *OfJNK* and *Ofp38* were sequenced by Sangon Biotech.

**TABLE 1:** Primers used in this study.

Primer	Sequence (5′–3′)
**Degenerate PCR**	
JNK-F	CAGATGCTGTGCGGCATCAA
JNK-R	TACTCCACCACCTCCTGGTA
P38-F	GGTGCTTACGGACAAGTTTG
P38-R	TCAAAGCTCTGATCGTAGGG
**RACE PCR**	
JNK 3RACE-F1	ATGGGCTACACGGAGAAC
JNK 3RACE-F2	GACATACTGTTCCCTAGCG
JNK 5RACE-R1	TTGTTCCACTGGTCGATGTG
JNK 5RACE-R2	GCCCAGGATCACCTCTGG
P38 3RACE-F1	GATCAGAGCTTTGAGGACAT
P38 3RACE-F2	CCTCAGCACATGAACACTG
P38 5RACE-R1	AAATCGGCTCCCATCAAAT
P38 5RACE-R2	AAGCTCACGGTAAGTCCT
**Real-time PCR**	
JNK-qF	ATGCGGGACAGAAGATCA
JNK-qR	GCAACTACGTGGAGAACC
P38-qF	TTGGTCTAGCACGTCCTACAGA
P38-qR	GTCAGCAGCTCAGCCATGATG
β-actin F	CCACACAGAACAGATGTATAAG
β-actin R	ATTCACTGCCAGCTTCATT
GAPDH-F	TTGAGGTTCAGGGTGACAAGG
GAPDH-R	CACCTTCCAAGTGAGCCGA

Based on the information obtained for the partially cloned *OfJNK* and *Ofp38* fragments, gene-specific primers for 5′ and 3′ rapid amplification of cDNA ends (RACE) were designed using Primer Premier 6.0 (Table 1). The 5′ and 3′ RACE-ready cDNAs were synthesized using the SMART^TM^ RACE cDNA amplification kit (TaKaRa) following the manufacturer’s instructions. RACE PCR was conducted under the following conditions: 25 amplification cycles at 94°C for 30 s and annealing at 60–70°C for 30 s (depending on the primer) and 72°C for 3 min. RACE PCR products were cloned into a linearized pRACE vector (a SMARTer^®^ RACE 5′/3′ Kit component) before sequencing. The complete *OfJNK* and *Ofp38* cDNA sequences were assembled using DNAMAN 6.0.

### Analysis of *OfJNK* and *Ofp38* Sequences

Using the open reading frame (ORF) finder^2^, we identified the ORFs of *OfJNK* and *Ofp38* and determined the amino acid sequences of the encoded proteins. The homology of *JNK* and *p38* was analyzed using the National Center for Biotechnology Information/Basic Local Alignment Search Tool database with other sequences available in GeneBank. The molecular weights and isoelectric points (pH[I]s) of the proteins were obtained using ProtParam^2^. Multiple sequence alignment was performed using DNAMAN 6.0, and a phylogenetic tree was created in MEGA 6.0 using the neighbor-joining method (Tamura et al., 2013) with a bootstrap test of 1,000 repetitions. Phosphorylation sites were predicted using NetPhos 2.0^3^, and protein domains or motifs were identified using SMART^4^.

### Quantitative Real-Time PCR

To analyze *OfJNK* and *Ofp38* expression levels, total RNA from different developmental stages, from various tissues, and under different environmental stresses of *O. furnacalis* was extracted and used to prepare cDNA templates using the PrimeScript RT Reagent Kit with gDNA Eraser (perfect real-time) (TaKaRa). The quantitative primers JNK-qF/JNK-qR and p38-qF/p38-qR were designed based on the *JNK* and *p38* sequences in *O. furnacalis*, and the internal reference gene primers actin-qF/actin-qR and GAPDH-qF/GAPDH-qR were designed according to the reported β*-*actin and *GAPDH* in the *O. furnacalis* DNA sequences (Table 1). Each quantitative real-time PCR (qRT-PCR) was conducted in a 20 μL reaction volume that comprised the following components: 10 μL ilaq^TM^ SYBR Green Supermix (2×), 1 μL cDNA template, 1 μL each primer (10 μM) (Table 1), and 7 μL ddH_2_O. RT-PCR was conducted using the Applied Biosystems 7500 RT-PCR system (Thermo Fisher) under the following conditions: denaturing at 95°C for 3 min followed by 39 cycles at 95°C for 30 s and 55°C for 30 s. To check the reproducibility of the experiment, qPCR for each treatment comprised three biological replications and three technical replications. The relative expression levels were quantified using the 2^–ΔΔCT^ method (Livak and Schmittgen, 2001).

### Statistical Analysis

All data were statistically analyzed using SPSS v. 20.0 (IBM Corporation, Armonk, NY, United States) and were compared using analysis of variance followed by the Tukey’s test. The data are presented as mean ± SEM. *P* < 0.05 was considered a statistically significant difference, which is clearly illustrated using different letters.

## Results

### Cloning *OfJNK* and *Ofp38* and Sequence Analysis

**FIGURE 1:**
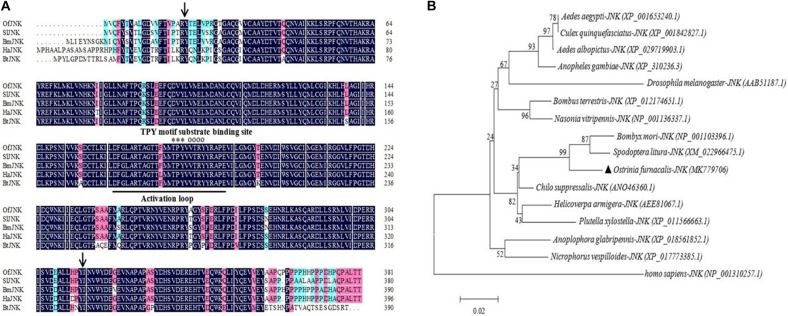
**(A)** Multiple sequence alignment of the amino acid sequences of *Ostrinia furnacalis* JNK with the JNK of other insects. In the representative JNK structure, the activation loop is underlined, substrate-binding site is indicated by empty circles, and threonine–proline–tyrosine (TPY) motif is indicated by asterisks found at positions 20–310 in the serine/threonine protein kinase (S_TKc) domain (black arrows). **(B)** The phylogenetic tree of *OfJNK* and *JNK* in various species. The location of *OfJNK* is indicated by a black-filled triangle. Abbreviations: Of, *Ostrinia furnacalis* (MK779706); Sl, *Spodoptera litura* (XM_022966475.1); Bm, *Bombyx mori* (NP_001103396.1); Ha, *Helicoverpa armigera* (AEE81067.1); Bt, *Bombus terrestris* (XP_012174651.1).

*OfJNK* and *Ofp38* were cloned using RT-PCR and RACE from *O. furnacalis* adults. We obtained 1,657 base pairs (bp) of the *OfJNK* cDNA (GenBank accession number MK779706) that contained a 472-bp 5′ untranslated region (UTR), 39-bp 3′ UTR, and 1,143-bp ORF encoding 381 amino acid residues; the molecular weight was calculated to be 43.44 kDa and theoretical pH(I) to be 6.06. Multiple sequence alignment showed that the *Of*JNK protein includes an active site, an activation loop structure, a substrate-binding site, and a threonine–proline–tyrosine motif (Figure 1A). The predicted serine/threonine protein kinase (S_TKc) domain was observed at positions 20–316. The identity of *Of*JNK compared with JNK in *S. litura*, *B. mori*, *H. armigera*, and *Bombus terrestris* was 90.7, 90.2, 81.16, and 88%, respectively (Figure 1A). Phylogenetic analysis showed that *Of*JNK was clustered with the JNK of other Lepidoptera and was most closely related to that of *H. armigera* and *S. litura* (Figure 1B).

**FIGURE 2:**
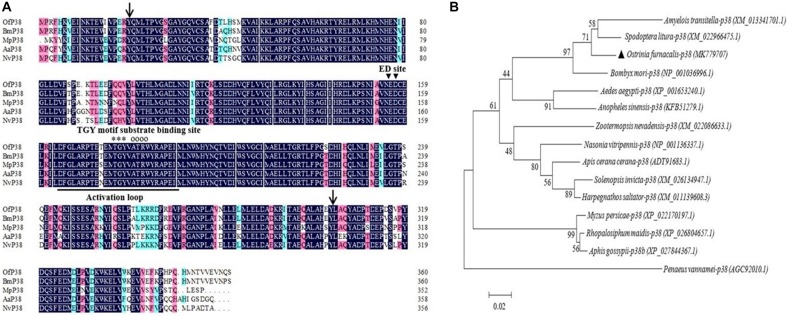
**(A)** Multiple sequence alignment of the amino acid sequences of *Ofp38* with the p38 from other insects. The predicted serine/threonine protein kinase (S_TKc) domain was found at positions 20–304 (black arrows). In the typical p38 structure, the activation loop is underlined, substrate-binding site is indicated by empty circles, extracellular signal-regulated kinase (ERK) and docking (ED) site is indicated by the black-filled inverted triangle, and threonine–glycine–tyrosine (TGY) motif is indicated by asterisks. **(B)** The phylogenetic tree of *Ofp38* and *p38* in various species. The location of *Ofp38* is indicated by a black-filled triangle. Abbreviations: Of, *Ostrinia furnacalis* (MK779707); Bm, *Bombyx mori* (NP_001036996.1); Mp, *Myzus persicae* (XP_022170197.1); Aa, *Aedes aegypti* (XP_001653240.1); Nv, *Nasonia vitripenn* (NP_001136337.1).

The 1,253 bp *Ofp38* cDNA (GenBank accession No.: MK779707) we obtained contains a 156-bp 5′ UTR, 17-bp 3′ UTR, and 1,080 bp ORF encoding 360 amino acid residues with a calculated molecular weight of 41.72 kDa and theoretical pH(I) of 5.84. Pairwise and multiple sequence alignments showed that *Ofp38* includes an active site, an activation loop structure, a specific threonine–glycine–tyrosine motif, a substrate-binding site ATRW, and an extracellular signal-regulated kinase docking (ED) site. The predicted S_TKc domain was observed at positions 20–304 (Figure 2A). The *Of*p38 protein showed a 94.75% similarity with *B. mori* p38, followed by that of *Nasonia vitripennis* (85.12%), *Aedes aegypti* (82.77%), and *Myzus persicae* (79.34%). Phylogenetic analysis revealed that the *Of*p38 protein was most homologous to that of *S. litura*, *B. mori*, and *Amyelois transitella*, which jointly constituted a correspondingly distinct clade (Figure 2B).

### Developmental Expression Profiles of Tissue-Specific *OfJNK* and *Ofp38*

**FIGURE 3:**
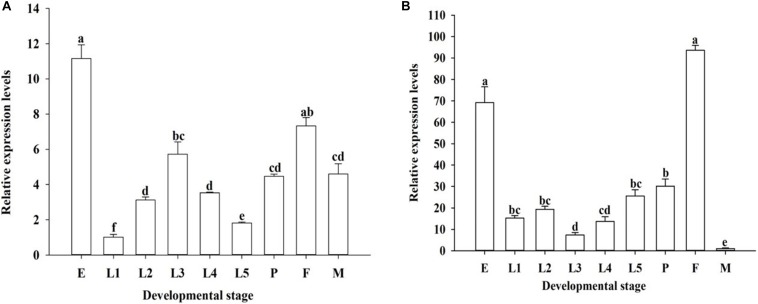
Relative expression levels of *OfJNK* **(A)** and *Ofp38* **(B)** in eggs (E), first to fifth instar nymphs (L1–L5), 3-day-old pupae (P), and 3-day-old adult female (F) and male (M). Data represent mean ± SEM (*N* = 3). The *OfJNK* expression level in the first instar (L1) was used for baseline; the *Ofp38* expression level in male (M) was used for baseline. Different letters above bars represent significant difference in relative expression levels (Tukey’s test; *P* < 0.05).

**FIGURE 4:**
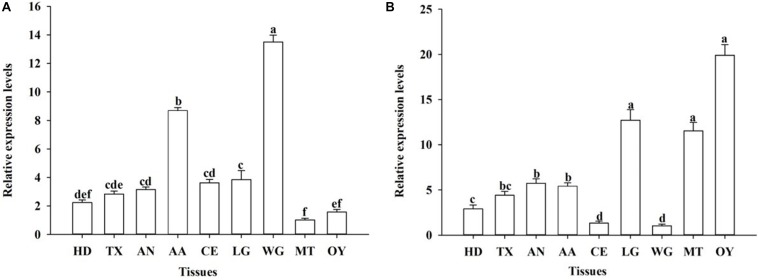
Relative expression levels of *OfJNK* (**A**) and *Ofp38* (**B**) in the head (HD) (without antennae and compound eye), thorax (TX), abdomen (AN), antenna (AA), compound eye (CE), leg (LG), wing (WG), midgut (MT), and ovary (OY) of mixed 10–30 adult females. Data represent mean ± SEM (*N* = 3). The *OfJNK* expression level in midgut (MT) was used for baseline; the *Ofp38* expression level in wing (WG) was used for baseline. Different letters above bars represent significant difference in relative expression levels (Tukey’s test; *P* < 0.05).

The developmental expression profiles of *OfJNK* and *Ofp38* were quantified in the insect’s eggs, first to fifth instar larvae, 3-day-old pupae, and 3-day-old adults (females and males). The results showed that *OfJNK* and *Ofp38* were expressed at all examined developmental stages (Figure 3). However, *OfJNK* was more highly expressed in the eggs than in the other developmental stages (Figure 3A), and the relative expression levels of *Ofp38* in 3-day-old adult females and eggs were significantly higher than those in the other developmental stages (Figure 3B). The results of tissue-specific expression showed that *OfJNK* was more highly expressed in the wings than in the other tissues (Figure 4A) and that the relative expression levels of *Ofp38* in the ovary were observably higher than those in the other tissues (Figure 4B).

### Expression of *OfJNK* and *Ofp38* After Exposure to Low Temperature

**FIGURE 5:**
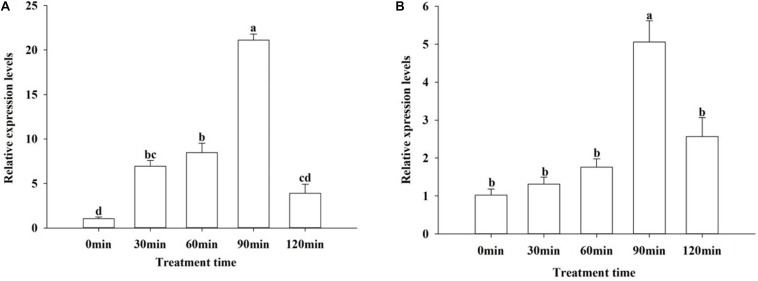
Relative expression levels of *OfJNK* (**A**) and *Ofp38* (**B**) at low temperature. Data represent mean ± SEM (*N* = 3). The *OfJNK* and *Ofp38* expression levels at 4°C treatment for 0 min (CK) were used for baseline. Different letters above bars represent significant difference in relative expression levels (Tukey’s test; *P* < 0.05).

To investigate whether the MAPKs are involved in the response of *O. furnacalis* to environmental stress, we measured the expression levels of *OfJNK* and *Ofp38* under low temperature stress. After exposing 3-day-old adults to 4°C for different durations, the relative expression levels of *OfJNK* and *Ofp38* were examined. The results showed that the expression levels first increased and eventually decreased with an increase in the treatment duration and that both *OfJNK* (Figure 5A) and *Ofp38* (Figure 5B) were highly expressed at 90 min.

### Effect of UV Irradiation on *OfJNK* and *Ofp38* Expression

**FIGURE 6:**
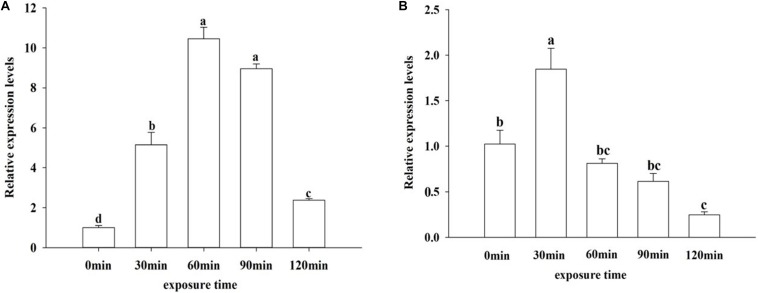
Relative expression levels of *OfJNK* (**A**) and *Ofp38* (**B**) after treatment with ultraviolet irradiation for different durations. Data represent mean ± SEM (*N* = 3). The *OfJNK* and *Ofp38* expression levels under UVA exposure for 0 min (CK) were used for baseline. Different letters above bars represent significant difference in relative expression levels (Tukey’s test; *P* < 0.05).

To examine the effect of UV irradiation on *OfJNK* and *Ofp38* expression, the insects were subjected to UVA exposure for different durations. Our results showed that the mRNA levels of *OfJNK* and *Ofp38* first increased and then decreased as the duration of UVA exposure increased and that *Ofp38* (Figure 6B) showed the highest expression levels at 30 min and *OfJNK* (Figure 6A) at 60 min.

## Discussion

Mitogen-activated protein kinases play vital roles in transmitting stress signals. Environmental stresses, such as cold, heat shock, UV irradiation, and viral and bacterial infections trigger these stress signals (Mizutani et al., 2003; Li et al., 2012). Previous studies on insects have shown that as important members of cell signaling pathways, JNK and p38 can be activated by various extracellular stresses such as UV irradiation, heat shock, pro-inflammatory factors, and specific antigens. Currently, the expression levels of *JNK* and *p38* have been well recorded under different environmental stressors, at different developmental stages, and in various tissue types in several insects. However, the information on *JNK* and *p38* in agricultural insects is limited. In our study, these two MAPK genes were cloned from *O. furnacalis* and were subsequently named *OfJNK* and *Ofp38*, respectively. Moreover, their molecular properties and expression levels were characterized under different environmental stimuli. The sequence and phylogenetic analyses showed a high sequence homology of the *Of*JNK and *Of*p38 proteins with the JNK and p38 proteins of *S. litura*, *B. mori*, *H. armigera*, and *N. vitripennis* (Figures 1A, 2A). Our study results suggested that the JNK and p38 signaling cascade systems may play an important role in the response of *O. furnacalis* to various environmental stresses (Xia et al., 1995; Ludwig et al., 2003).

Using qPCR, we observed that *OfJNK* and *Ofp38* were expressed in the developmental stages of *O. furnacalis* but that their expression levels were significantly different depending on the specific stage. *OfJNK* was highly expressed in the eggs compared with those in the other developmental stages. This is similar to previous study results that the JNK signaling pathway was involved in egg formation in *Drosophila* (Suzanne et al., 2001). The relative expression levels of *Ofp38* in eggs and 3-day-old adult females were significantly higher than those in the other developmental stages. This difference might be related to the changes made by the p38 signaling pathway to the expression levels of this gene through the phosphorylation of transcription factors at different developmental stages of the insect, thereby allowing it to participate in cell growth and proliferation (Li et al., 2003). Tissue-specific quantitative results showed that *OfJNK* and *Ofp38* were expressed in various *O. furnacalis* tissues, which is consistent with the study results on mammalian JNK and p38 reported by Widmann et al. (1999). The expression levels of *OfJNK* were higher in the wings than in the other tissues. This corresponds to the results of previous studies in which the JNK signaling pathway reportedly participated in wing formation and development of the insect (Zhang et al., 2019). Similarly, we found that *Ofp38* was significantly higher in the ovary than in the other tissues, indicating that *Ofp38* may be involved in the process of ovarian cell growth and development in *O. furnacalis* (Lu et al., 2007; Sheng et al., 2015).

Furthermore, we detected the expression levels of *OfJNK* and *Ofp38* in *O. furnacalis* under low temperature and UVA irradiation. Low temperature and UV irradiation are important stress factors that impact every living creature including insects. Some studies have reported that *JNK* and *p38* could respond to various environmental stresses, such as cold stress, heat shock, oxidative stress, immunostimulants, serum starvation, and UV irradiation, in cells (Hanks and Hunter, 1995; Han et al., 1998). In the present study, the expression levels of *OfJNK* and *Ofp38* significantly increased when *O. furnacalis* adults were exposed to 4°C for 90 min, which indicated that JNK and p38 might be involved in responses to low temperatures. Insects can feel signals and transmit them to the cellular machinery through signal transduction to regulate gene expression. We suspected that JNK/P38 pathway might be activated by low temperatures, which could promote DNA damage repair and cell growth in *O. furnacalis* to resist environmental stress. This result is consistent with a report that JNK is activated to enhance DNA repair and promote cell survival in DNA damage response and the activated p38 pathway mediates cell growth and proliferation (Johansson et al., 2000; Chock et al., 2010). However, as time elapsed, the JNK and p38 signals were attenuated. It may be due to the fact that prolonged stress beyond the maximum tolerance of the JNK/P38 pathway leads to cell damage. It is reported that short-term activation of JNK and p38 can promote cell survival, while sustained activation can cause cell apoptosis (Harper and Lograsso, 2001). This has been founded in *S. crassipalpis* and the liver and kidneys of the turtles (Greenway and Storey, 2000; Fujiwara and Denlinger, 2007). UV-A light used as light sources in light traps is regarded as an environmental stress factor to insects (Zhang and Meng, 2018). In our study, when *O. furnacalis* adults were exposed to UVA, the expression levels of *Ofp38 and OfJNK* significantly increased at 30 and 60 min, respectively, which indicated that JNK and p38 might be involved in responses to UVA exposure. We suspected that JNK/P38 pathways could be activated UV-A irradiation, which in turn helps tolerate UV stress and improves the ability of *O. furnacalis* to adapt to stress. However, with exposure to UV light for longer time (120 min), the expression levels of *OfJNK* and *Ofp38* decreased. This result agrees with findings of Liu et al. (2019) who reported that UVA stress induced mRNA expression of JNK in *H. armigera* adult females and that the highest expression level was at 60 min, which further decreased with the exposure time.

## Conclusion

In conclusion, the results of this study suggested that *OfJNK* and *Ofp38* responded to low temperature and UV irradiation, and that the MAPK signaling pathway plays critical roles in response to environmental stresses in *O. furnacalis*. We believe these results will broaden our knowledge regarding the MAPK signaling pathway in insects.

## Data Availability Statement

Publicly available datasets were analyzed in this study. This data can be found in GenBank under the accession numbers MK779706 and MK779707.

## Author Contributions

C-YZ conceived and designed the experiments. LS cloned and analyzed *OfJNK* and *Ofp38*, examined *OfJNK* and *Ofp38* expression levels, and prepared the manuscript. All authors finalized the manuscript, and have read and approved the final manuscript.

## Conflict of Interest

The authors declare that the research was conducted in the absence of any commercial or financial relationships that could be construed as a potential conflict of interest.

## Footnotes

1 http://kr.expasy.org/tools/protparam.html

2 http://www.ncbi.nlm.nih.gov/orffinder/

3 http://www.cbs.dtu.dk/services/NetPhos/

4 http://smart.embl-heidelberg.de
